# Hypoxia as a target for drug combination therapy of liver cancer

**DOI:** 10.1097/CAD.0000000000000516

**Published:** 2017-05-24

**Authors:** Cressida Bowyer, Andrew L. Lewis, Andrew W. Lloyd, Gary J. Phillips, Wendy M. Macfarlane

**Affiliations:** a School of Pharmacy and Biomolecular Sciences, University of Brighton, Lewes Road, Brighton; b Biocompatibles UK Ltd, Camberley, UK

**Keywords:** doxorubicin, HepG2, hypoxia, mTOR, rapamycin, transarterial chemoembolization

## Abstract

Hepatocellular carcinoma (HCC) is the third most frequent cause of cancer deaths worldwide. The standard of care for intermediate HCC is transarterial chemoembolization, which combines tumour embolization with locoregional delivery of the chemotherapeutic doxorubicin. Embolization therapies induce hypoxia, leading to the escape and proliferation of hypoxia-adapted cancer cells. The transcription factor that orchestrates responses to hypoxia is hypoxia-inducible factor 1 (HIF-1). The aim of this work is to show that targeting HIF-1 with combined drug therapy presents an opportunity for improving outcomes for HCC treatment. HepG2 cells were cultured under normoxic and hypoxic conditions exposed to doxorubicin, rapamycin and combinations thereof, and analyzed for viability and the expression of hypoxia-induced HIF-1α in response to these treatments. A pilot study was carried out to evaluate the antitumour effects of these drug combinations delivered from drug-eluting beads *in vivo* using an ectopic xenograft murine model of HCC. A therapeutic doxorubicin concentration that inhibits the viability of normoxic and hypoxic HepG2 cells and above which hypoxic cells are chemoresistant was identified, together with the lowest effective dose of rapamycin against normoxic and hypoxic HepG2 cells. It was shown that combinations of rapamycin and doxorubicin are more effective than doxorubicin alone. Western Blotting indicated that both doxorubicin and rapamycin inhibit hypoxia-induced accumulation of HIF-1α. Combination treatments were more effective *in vivo* than either treatment alone. mTOR inhibition can improve outcomes of doxorubicin treatment in HCC.

## Introduction

Hepatocellular carcinoma (HCC) is the fifth most common cancer worldwide and the third most common cause of cancer-related deaths 1; it is often diagnosed at an intermediate or an advanced stage, when treatment options are limited and the prognosis is poor. Transarterial chemoembolization (TACE) using the anthracycline antibiotic doxorubicin is the standard treatment for unresectable intermediate HCC 2. TACE combines embolization with locoregional delivery of chemotherapeutic agent(s). HCCs are typically highly vascularized 3 and embolization therapies for HCC exploit the fact that the liver tumour is fed by the hepatic artery, whereas the normal liver tissue is fed mainly by the portal vein 4. Disruption of the arterial blood supply results in a depletion of oxygen and nutrients to the tumour cells, and consequent necrosis and tumour shrinkage. Drug-eluting bead transarterial chemoembolization (DEB-TACE) is a refinement of TACE and provides a one-step procedure for both embolization and drug delivery. DEB-TACE enables a controlled, localized and sustained release of the drug to the tumour bed, with reduced systemic doxorubicin and an improved safety profile compared with cTACE 5–7. Currently, the most widely used and studied DEB is DC Bead, a sulphonate-modified polyvinyl alcohol hydrogel microsphere system that can be conveniently loaded in the hospital pharmacy with cationic drugs such as doxorubicin hydrochloride 8. Once the drug has been fully sequestered into the beads by an ion-exchange process, it is delivered to the interventional radiologist and administered through the hepatic arterial vasculature to the site of the tumour, where the drug is then released in a controlled and sustained manner over the next weeks. A feature of the embolization of the blood vessels is the generation of ischaemia, which is the primary cause of tumour cell death following this procedure, but that also may have downstream ramifications.

Tumour hypoxia is a feature of all solid tumours, and is a requirement for tumour growth 9. Intratumoural hypoxia and the hypoxic phenotype are implicated in therapy resistance, tumour malignancy, tumour progression and a poor prognosis. A negative, but inevitable, consequence of embolization therapy is the de novo formation of hypoxic regions within the tumour. Hypoxic tumour cells undergo phenotypic adaptations that allow the cells to survive and results in the clonal selection of refractory tumour cells 10. The transcription factor, hypoxia-inducible factor 1 (HIF-1) is the master regulator of cellular responses to hypoxia. HIF-1 is a heterodimeric DNA-binding complex consisting of α and β subunits. The HIF-1α subunit is subjected to oxygen-dependent post-translational degradation by hydroxylation of prolyl residues within the oxygen-dependent degradation domain, which promotes the interaction of HIF-1α with the von Hippel–Lindau tumour-suppressor protein, a component of the protein–ubiquitin ligase complex that targets HIF-1α for rapid degradation. Under hypoxic conditions, HIF-1α stabilizes and translocates to the nucleus, where it heterodimerizes with HIF-1β. The HIF-1 complex binds to hypoxia response elements present in the enhancer or promoter regions of the HIF-1 target gene 11. The transcriptional activity if HIF-1 is subjected to oxygen-dependent regulation, whereby the asparagyl hydroxylase factor inhibiting HIF-1 (FIH-1) blocks the association of transcriptional coactivators CREB (cAMP response element binding)-binding protein/p300 with the C-terminal transactivation domain of HIF-1α in the presence of oxygen 12. Clinical and animal studies have reported increased HIF-1α after TACE in both plasma and tumour samples 13–15. The genes regulated by HIF-1 enable the cells to survive in a hypoxic environment and thus promote tumour growth 16.

The cytotoxicity of doxorubicin against cancer cells is attributed to the intercalation of the drug in the DNA of dividing cells; the induction of topoisomerase-II-mediated strand breaks; and the generation of oxygen radicals that damage DNA and cell membranes 17–19. Recent data suggest that doxorubicin inhibits the transcription of HIF-mediated genes by blocking the binding of HIF-1 to the promoter region of hypoxia response genes 20.

Rapamycin interferes with the PI3K/Akt/mTOR signalling pathway, a pathway that is known to play an important role in cancer progression and is known to be deregulated in around half of all HCCs 21. Activation of mTOR (mechanistic target of rapamycin) increases the rate of translation of HIF-1α 22. The mTOR inhibitor rapamycin has been shown to exert antitumoural effects *in vitro* and *in vivo* 23–30. In addition to the use of either doxorubicin or rapamycin against HCC, there is also potential for the use of both in combination. Drugs that target molecular pathways exert their effects on stromal tissues and cells and the processes that support tumour growth, as well as on tumour cells themselves. Rapamycin and doxorubicin have been shown to have additive effects *in vivo* in murine models of liver, prostate, cervical and lung cancers 31–33.

The majority of in-vitro investigations are carried out at ambient (21%) oxygen, and do not properly model in-vivo intratumoural physiology, wherein oxygen concentrations are likely to be much lower 34. To understand the biology of cells occupying hypoxic niches, in-vitro research needs to be carried out at physiologically relevant oxygen concentrations. Increased understanding of the pathophysiological responses to hypoxia will contribute towards improved treatment regimens and better outcomes for patients.

Here, we established a hypoxic model of HCC by cultivating HepG2 cells at 1% oxygen. The time-dependent and concentration-dependent effects of doxorubicin, rapamycin and both drugs in combination on the viability of HepG2 cells were investigated under both normoxic and hypoxic culture conditions. SDS-PAGE and Western Blotting were then used to evaluate the responses of hypoxia-induced HIF-1α after application of the same chemotherapeutics. Finally, the antitumour effects of DEBs loaded with doxorubicin, rapamycin and both drugs in combination were investigated *in vivo* using an ectopic xenograft murine model of HCC.

## Materials and methods

### Cell lines

HepG2 cells were obtained from the American Type Culture Collection (Rockville, Maryland, USA) and cultured as recommended by the supplier. Cells were maintained in minimum essential medium+Earle’s salts+l-glutamine, supplemented with 10% foetal bovine serum heat-inactivated and 1% nonessential amino acids (all from PAA Laboratories, GmBH, Cölbe, Germany). For hypoxic incubations (1% oxygen), the cells were cultured in a hypoxic glove box (COY Laboratory Products Inc., Grass Lake, Michigan USA).

### Cell viability assay

HepG2 cells were seeded onto 96-well plates (1×10^4^ cells/well in 200 µl aliquots) and incubated overnight. Plates were exposed to doxorubicin (Zhejiang Hisun Pharmaceuticals, Zhejiang, China), rapamycin (LC Laboratories, Woburn, Massachusetts, USA) or a combination of both and incubated for 24, 48 and 72 h under normoxic or hypoxic conditions (six replicate wells for each drug concentration). Cell viability was assessed using the 3-(4, 5 dimethylthiazol-2-yl)-5-(3-carboxymethoxyphenyl)-2-(4 sulphophenyl) 2H-tetrazolium, inner salt (MTS) cytotoxicity assay (Promega, Southampton, UK) according to the manufacturer’s instructions.

### Western blot analysis

Cells were harvested and nuclear extracts were prepared. Overall, 20 µg of protein (as determined by Bradford assay) was resolved on a 10% polyacrylamide gel and transferred to a polyvinylidene fluoride membrane (Immobilon-P membrane; Millipore Corp., Watford, Hertfordshire). Immunoblotting was carried out using antibodies against HIF-1α (Cell Signalling Technology, Danvers, Massachusetts, USA) and Lamin B1 (Abcam, Cambridge, UK). Protein was detected and quantified using chemiluminescence (Amersham ECL Plus Western Blotting Detection Reagents; GE Healthcare, Buckinghamshire, UK) and FlouroChem software (Alpha Innotech; San Leandro, California, USA); the target protein was normalized to a house-keeping protein control.

### Immunohistochemistry

HepG2 cells were seeded onto chamber slides (Fisher Scientific, Leicestershire, UK) and incubated under normoxic conditions until confluence was 60%. The cells were then incubated in either 21% oxygen or 1% oxygen for 24 h and fixed with 3.7% formalin. The cells were incubated overnight with antibodies to HIF-1α, and then incubated with tetramethylrhodamine-5-(and 6)-isothiocyanate fluorescein isothiocyanate conjugated secondary antibody. 4′,6-Diamidino-2-phenylindole staining was used to visualize the nucleus. Images are taken at ×100 magnification.

### Ectopic xenograft murine model of hepatocellular carcinoma

Experiments were conducted by EPO-GmbH and approved by LAGeSo (State Office of Health and Social Affairs), Berlin. National Medical Research Institute nu/nu mice (Taconic M&B, Ry, Denmark), adult females of 20 g were randomly assigned to treatment groups. In all, 1×10^7^ HepG2 cells from the culture were transplanted subcutaneously at day 0. Treatment started when tumours were of palpable size. DC Bead microspheres (Biocompatibles UK Ltd, Camberley, UK) were loaded to a doxorubicin concentration of 25 mg/ml, a rapamycin concentration of 20 mg/ml and a combination of both as described previously 35. The beads were lyophilized and gamma sterilized (Isotron PLC, Wiltshire, UK). A 3 ml aliquot of alginate solution (CellMed Ag, Alzenau, Germany) was added to a vial containing 1 ml of beads and mixed. Overall, 100 μl of bead/alginate mix was applied adjacent to the tumour by direct injection. For oral administration, rapamycin powder was dissolved in ethanol to a concentration of 10 mg/ml and diluted to 0.01 mg/ml in drinking water. A dose of 1 mg/kg/day was administered by gavage. Tumour growth was measured twice per week in two perpendicular diameters using a calliper. Tumour volume was calculated using the ellipsoid volume formula (volume=*π*/6×length×width^2^). The mice were inspected for signs of toxicity and behavioural changes immediately after application of the beads and then twice daily. Body weight change was used as a parameter for toxicity and was determined twice per week. Mice were euthanized at a moribund stage or if the tumour was larger than 10% of the total body weight. The tumours were excised and weighed postmortem.

## Results

### The effects of doxorubicin, rapamycin and combinations on cell viability under normoxic and hypoxic conditions

**Fig. 1:**
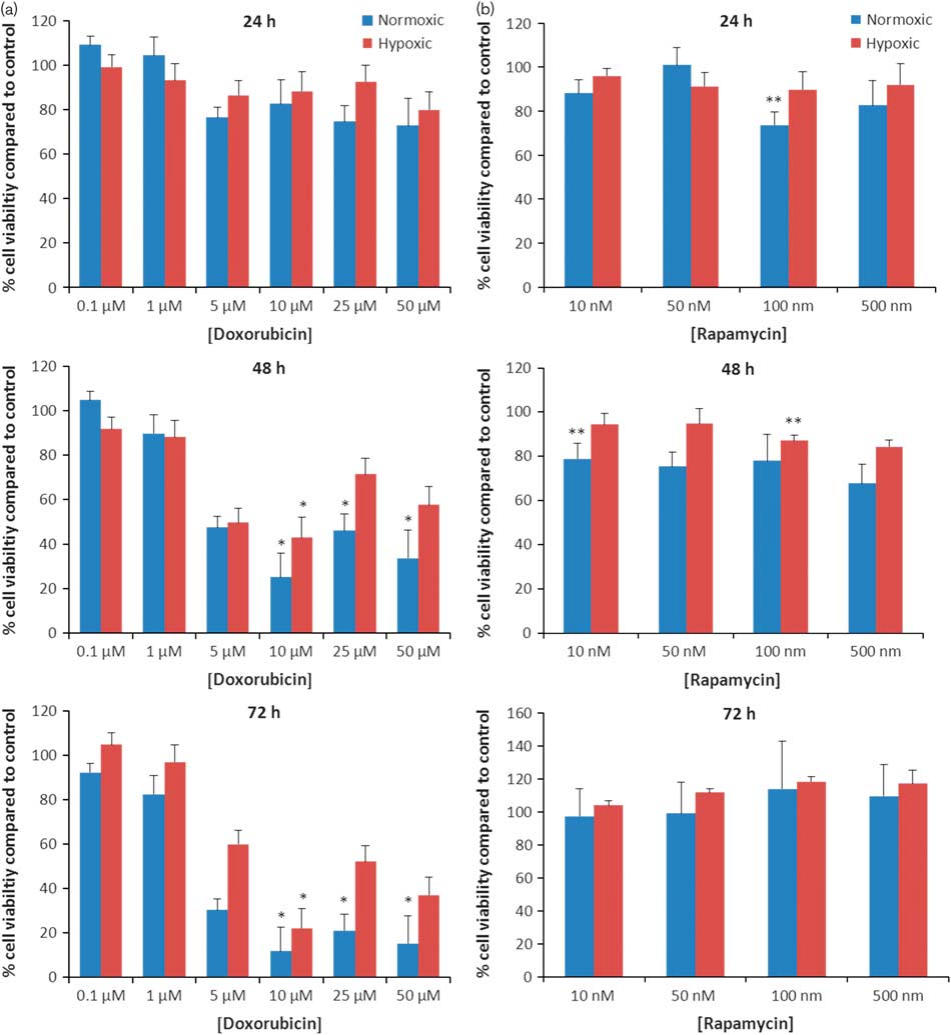
The effects of (a) doxorubicin and (b) rapamycin on the viability of HepG2 cells cultured under normoxic and hypoxic conditions. HepG2 cells were seeded onto 96-well plates (1×10^4^ cells/well) and incubated overnight. Plates were then exposed to doxorubicin or rapamycin and incubated for 24, 48 and 72 h under normoxic or hypoxic conditions. Cell viability was estimated using the MTS assay and normalized to untreated control. Data points represent mean±SEM from at least three independent experiments. For statistical analysis of doxorubicin treatments, analysis of variance with Tamhane post-hoc comparisons was used; * denotes a significant decrease in cell viability compared with the control *P*<0.01. For statistical analysis of rapamycin treatment, a one-tailed *t*-test was carried out; ** denotes a significant decrease in cell viability compared with the control *P*<0.05.

To determine the effect of doxorubicin, rapamycin and combinations of both on HepG2 cell viability, a series of time-dependent and concentration-dependent MTS assays were carried out. The effects of doxorubicin alone are summarized in Fig. 1a. After 48 h (Fig. 1a), 10, 25 and 50 µmol/l doxorubicin treatments all reduced the viability of HepG2 cells cultured under normoxic conditions (*P*=0.000, 0.002 and 0.004, respectively). The 10 µmol/l treatment was not significantly more effective than either the 25 or the 50 µmol/l treatments (*P*=0.126 and 0.648, respectively). After 72 h, 10, 25 and 50 µmol/l doxorubicin treatments all reduced the viability of HepG2 cells cultured under normoxic conditions (*P*=0.000, 0.001 and 0.010, respectively). The 10 µmol/l treatment was not significantly more effective than either the 25 or the 50 µmol/l treatments (*P*=0.993 and 1.000, respectively). The hypoxic cells were more resistant to doxorubicin treatment. After 48 h, only the 10 µmol/l treatment significantly reduced the cell viability of HepG2 cells cultured under hypoxic conditions (*P*=0.005); treatments of 25 and 50 µmol/l had no significant effect (*P*=0.280 and 0.546, respectively). After 72 h, only the 10 µmol/l treatment significantly reduced the cell viability of HepG2 cells cultured under hypoxic conditions (*P*=0.001); treatments of 25 and 50 µmol/l had no significant effect (*P*=0.288 and 0.065, respectively).

**Fig. 2:**
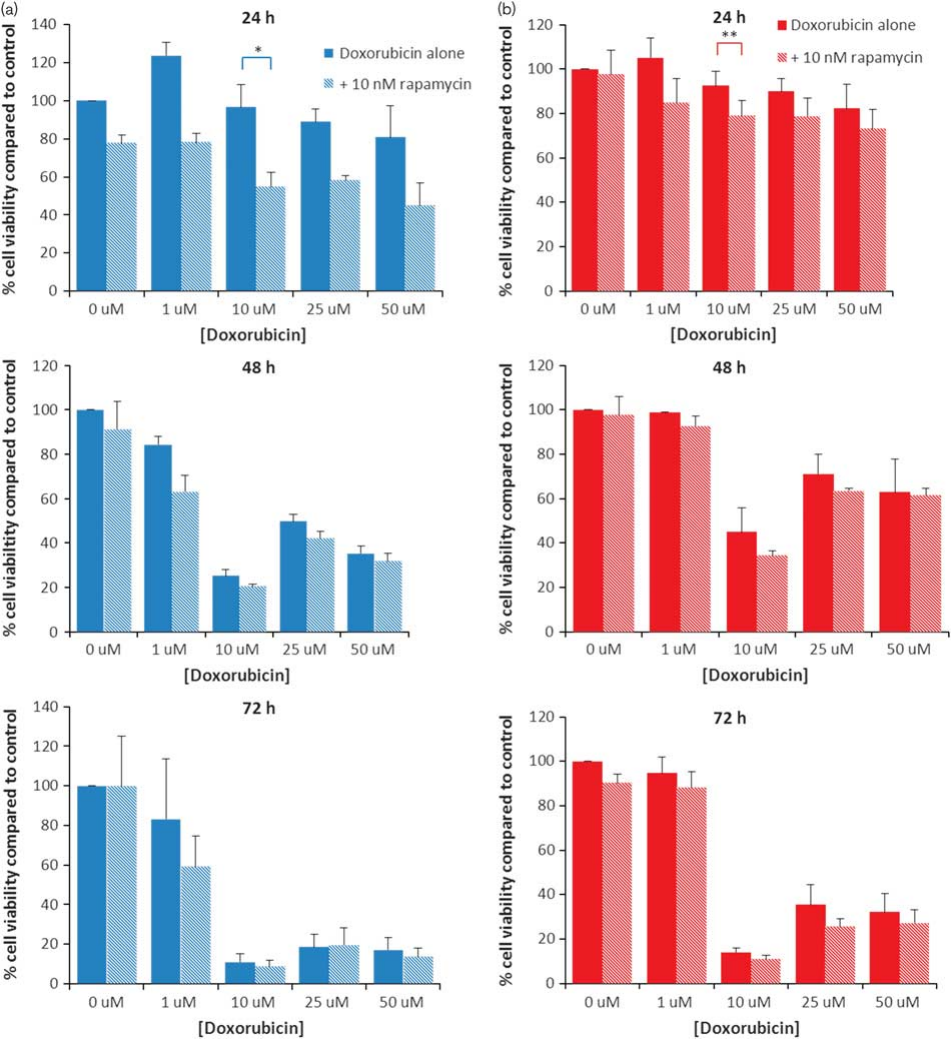
The effects of doxorubicin+10 nmol/l rapamycin on the viability of HepG2 cells cultured under (a) normoxic and (b) hypoxic conditions. HepG2 cells were seeded onto 96-well plates (1×10^4^ cells/well) and incubated overnight. Plates were then exposed to doxorubicin+10 nmol/l rapamycin and incubated for 24, 48 and 72 h under normoxic or hypoxic conditions. Cell viability was estimated using the MTS assay and normalized to untreated control. Data points represent mean±SEM from at least three independent experiments. For statistical analysis, analysis of variance was carried out. *P<0.000, **P<0.05.

The effects of rapamycin alone are summarized in Fig. 1b. The lowest effective concentration of rapamycin as a single agent after 24 h was 100 nmol/l for normoxic cells (*P*=0.011). Hypoxic cells were resistant to concentrations up to 500 nmol/l. The lowest effective concentration of rapamycin as a single agent after 48 h was 10 nmol/l for normoxic cells (*P*=0.028) and 100 nmol/l for hypoxic cells (*P*=0.006). Analysis of variance found a significant increase in cytotoxicity between doxorubicin alone and doxorubicin+10 nmol/l rapamycin combinations in both normoxic and hypoxic cells after 24 h (*P*=0.000 and 0.05, respectively), but this effect was not observed at the later time points (Fig. 2).

### Effects of doxorubicin and rapamycin on HIF-1α induction

**Fig. 3:**
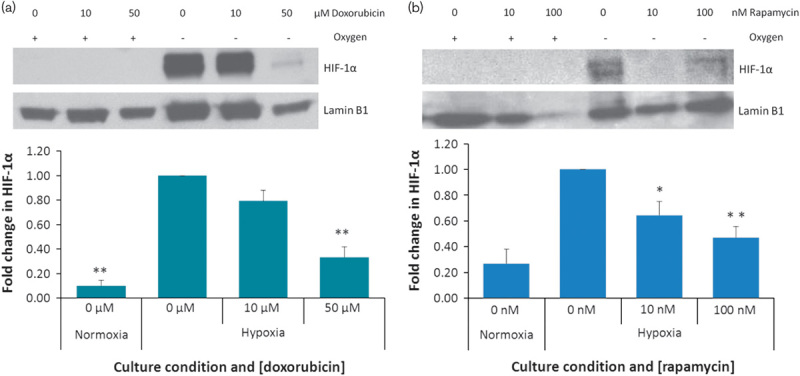
Nuclear accumulation of hypoxia-inducible factor 1α (HIF-1α) after doxorubicin and rapamycin treatments. Normoxic and hypoxic HepG2 cells were exposed to doxorubicin or rapamycin for 24 h. Nuclear extracts were fractionated on a 10% SDS-PAGE gel, transferred to a PVDF membrane and probed with anti-HIF-1α antibodies. Proteins were visualized using chemiluminescence. The membrane was stripped and reprobed using antibodies against the nuclear house-keeping protein Lamin B1. Protein levels were quantified using densitometry analysis. HIF-1α was normalized to Lamin. Fold change compared with untreated hypoxic cells was calculated. Statistical analysis was carried out using a *t*-test. *P<0.05, **P<0.01.

**Fig. 4:**
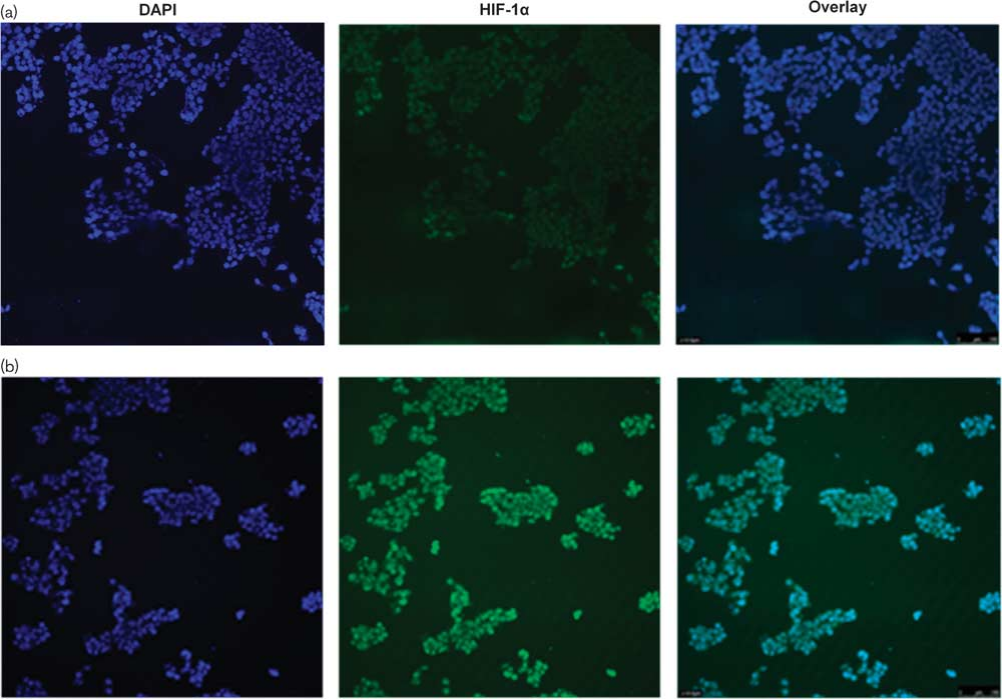
Immunohistochemistry staining for hypoxia-inducible factor 1 (HIF-1) in normoxic and hypoxic HepG2 cells. Cells were seeded onto chamber slides. HepG2 cells were seeded onto chamber slides and incubated under normoxic conditions until confluence was 60%. The cells were then incubated under either (a) normoxic conditions or (b) hypoxic conditions for 24 h. The cells were incubated overnight with antibodies to HIF-1α and then incubated with the TRITC-conjugated secondary antibody. 4′,6-Diamidino-2-phenylindole staining was used to visualize the nucleus. Images are taken at ×100 magnification. HIF-1α is absent in normoxic cells and present in hypoxic cells.

To determine the effect of these agents on the hypoxic induction of HIF-1α, SDS-PAGE and Western Blotting were carried out on nuclear extracts from HepG2 cells exposed to doxorubicin or rapamycin when cultured under normoxic or hypoxic conditions. As shown in Fig. 3, 24 h exposure to hypoxic conditions resulted in a significant increase in nuclear HIF-1α (*P*=0.001). This was confirmed using immunohistochemistry (Fig. 4). Although the doxorubicin treatment of 10 µmol/l had no significant effect, the 50 µmol/l treatment significantly reduced the amount of HIF-1α detected in the nucleus (*P*=0.002) (Fig. 3a). Doxorubicin treatment did not result in the induction of HIF-1α in the nucleus of normoxic cells (Fig. 3a). Both the 10 nmol/l rapamycin and the 100 nmol/l rapamycin treatments significantly inhibited the accumulation of HIF-1α in the nucleus of hypoxic cells (*P*=0.021 and 0.001, respectively) (Fig. 3b).

### Effects of doxorubicin and rapamycin drug-eluting beads in a murine model of hepatocellular carcinoma

**Fig. 5:**
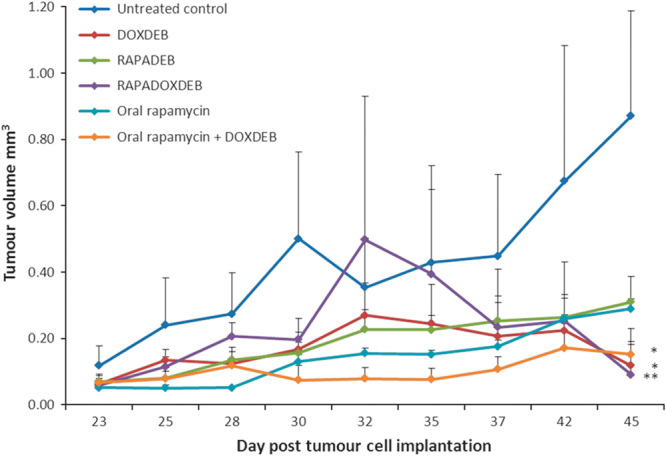
Antitumoural activity of doxorubicin and rapamycin treatments in a mouse model of hepatocellular carcinoma. In all, 5×10^6^ HepG2 cells were subcutaneously implanted in NMRI: nu/nu mice (day 0). Tumour was palpable at day 23 after implantation and treatment was initiated. Rapamycin was administered by gavage at a dose of 1 mg/kg/day. 100 µl of beads loaded as specified was injected adjacent to the tumour. Tumour volume was measured at days 23, 25, 28, 30, 32, 35, 37, 42 and 45. Data shown represent the mean value of three replicates per group±SEM, apart from the control group, where data represent the mean of two replicates±SEM. Statistical analysis was carried out using analysis of variance analysis. *P≤0.05; **P≤0.01. DOXDEB, doxorubicin-loaded beads; RAPADEB, rapamycin-loaded beads; RAPADOXDEB, doxorubicin and rapamycin co-loaded beads.

**Fig. 6:**
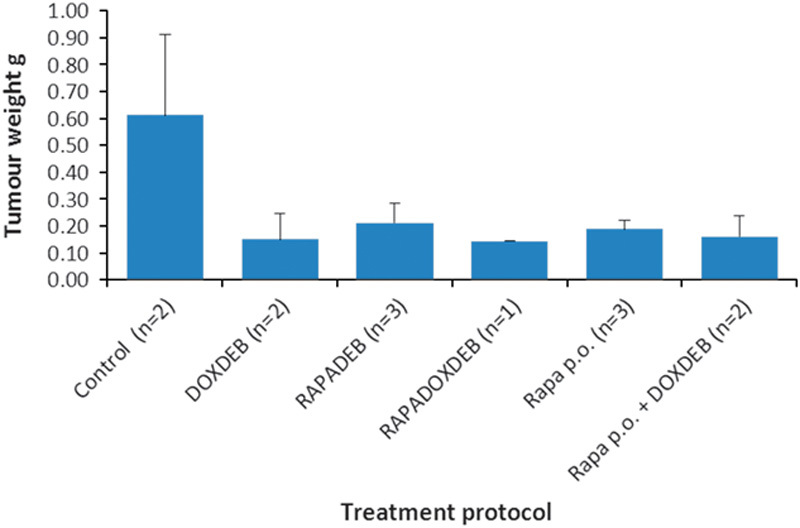
Effects of doxorubicin and rapamycin treatments on tumour weight at day 45 in a mouse model of hepatocellular carcinoma. Mice were euthanized at day 45, and the tumours were excised and weighed. Data represent mean±SEM. Because of the small sample sizes, statistical analysis was not carried out. DOXDEB, doxorubicin-loaded beads; p.o., orally; RAPADEB, rapamycin-loaded beads; RAPADOXDEB, doxorubicin and rapamycin co-loaded beads.

Consequent to the findings described above, the antitumour effects of doxorubicin and rapamycin, both as monotherapies and in combination, were evaluated *in vivo* in a pilot study using a murine model of HCC (Fig. 5). HepG2 tumour-bearing mice were treated with doxorubicin-loaded beads, rapamycin-loaded beads, rapamycin and doxorubicin co-loaded beads, oral rapamycin and doxorubicin-loaded beads in combination with oral rapamycin. At day 45, analysis of variance showed a significant difference between the treatment groups (*P*=0.008). Post-hoc comparisons indicated that three treatment regimens resulted in a significant reduction in tumour volume compared with the control – doxorubicin-loaded beads (*P*=0.014), rapamycin and doxorubicin co-loaded beads (*P*=0.011) and oral rapamycin in combination with doxorubicin-loaded beads (*P*=0.021). Rapamycin monotherapies inhibited tumour growth for the duration of the experiment and oral rapamycin inhibited tumour growth more effectively than rapamycin-loaded beads, although the pattern of growth inhibition was the same for both treatments. Doxorubicin-loaded beads inhibited tumour growth in a manner similar to rapamycin-loaded beads up to day 32. We found that doxorubicin was necessary and sufficient for tumour regression, which occurred from day 32 in the doxorubicin-loaded bead cohort and in the rapamycin and doxorubicin co-loaded bead cohort. The most effective treatment overall was the combination of doxorubicin-loaded beads and oral rapamycin, which resulted in almost total inhibition of tumour growth to day 35, tumour regression from day 28 to 30 and day 42 to 45 and complete tumour destruction reported in one animal by day 45. All treatments resulted in decreased tumour weight compared with the control (Fig. 6). There was evidence of increased antitumoural activity with combination therapies compared with either treatment alone, and all treatments were well tolerated.

## Discussion and conclusions

Here, we have identified a doxorubicin concentration of 10 µmol/l that is effective against normoxic and hypoxic HCC cells. This 10 µmol/l dose is commensurate with concentrations of doxorubicin that are eluted from DC Beads at distances of up to 350 and 600 µm 36–38. Viable hypoxic cells have been observed at distances of 50–250 µm from the nearest blood vessel 39; thus, tissue penetration of doxorubicin is sufficient to target hypoxic cells.

Hypoxia-induced mechanisms of resistance to doxorubicin are likely to be multifactorial and include reduced drug accumulation and increased drug efflux 40–42, resistance to apoptosis 43, decreased levels of topoisomerase II 44,45 and a reduction in free radical-dependent DNA damage 46. Toxic effects that are oxygen dependent are reduced in hypoxia 47.

Hypoxia protected cells from doxorubicin-induced cytotoxicity (48 and 72 h treatments) at concentrations 10, 25 and 50 μmol/l. The cytotoxicity of doxorubicin is because of a number of different mechanisms, with the activation of one mechanism potentially having an inhibitory effect on other mechanisms and particular co-factors being required for efficient cytotoxicity in some cases. For example, doxorubicin is a topoisomerase II poison with the intercalation of doxorubicin into cellular DNA at higher drug concentrations itself potentially inhibiting the binding of TOP2 to cellular DNA and decreasing TOP2-mediated cytotoxicity 48. The complex interplay of these factors with hypoxia-induced cell survival mechanisms could explain why a 10 μmol/l dose is more effective than higher doses. In this study, only the 50 μmol/l dose of doxorubicin was shown to inhibit hypoxia-induced nuclear accumulation of HIF-1α. In line with this study, Potmesil *et al.* 49 reported that the frequency of single-strand DNA breaks in mouse leukaemia cells increased with doses of doxorubicin between 0.2 and 20 μmol/l, then plateaued in normoxic cells and declined in hypoxic cells until the concentration was increased to 200 μmol/l. Extracellular pH levels are also higher in hypoxic cells compared with normoxic cells because of the Warburg effect, which reduces the proportion of doxorubicin in the nonionized membrane-permeable form and consequently causes a reduction in the accumulation of doxorubicin into cells 50–52. Different local doxorubicin concentrations will also impact on this drug-partitioning effect and drug-efflux mechanisms may also be more pronounced when the drug is at higher concentrations.

We subsequently established that 10 nmol/l rapamycin inhibited cell viability in normoxic cells, but that a concentration of 100 nmol/l was required to inhibit cell viability in hypoxic cells. Combinations of 10 nmol/l rapamycin with doxorubicin were more effective against both normoxic and hypoxic HepG2 cells than either treatment alone. A primary mechanism contributing towards drug resistance is increased drug efflux because of the upregulation of P-glycoprotein MDR1 40,41. Rapamycin has been reported to improve the uptake of chemotherapeutics in multidrug-resistant cell lines because of competitive inhibition as a result of a direct interaction of rapamycin with Pgp 53–55.

To ascertain the possible effects of doxorubicin and rapamycin on responses to hypoxia-induced survival pathways, we investigated their effects on hypoxia-induced nuclear accumulation of HIF-1. Doxorubicin of 50 µmol/l and rapamycin of 10 and 100 nmol/l attenuated the stabilization of HIF-1α under hypoxic conditions.

Consequent to the findings described above, the antitumour effects of doxorubicin and rapamycin, both as monotherapies and in combination *in vivo*, were evaluated in a murine model of HCC. Rapamycin monotherapies inhibited tumour growth for the duration of the experiment. Doxorubicin was necessary and sufficient for tumour regression. The most effective treatment overall appears to be the combination of DOXDEB and oral rapamycin, which resulted in almost total inhibition of tumour growth to day 35, tumour regression from day 28 to 30 and day 42 to 45 and complete tumour destruction reported in one animal by day 45. There was evidence of increased antitumoural activity with combination therapies compared with either treatment alone, and all treatments were well tolerated. In agreement with our findings, combinations of doxorubicin with mTOR inhibition were found to be more effective than monotherapies in an orthotopic syngeneic rat HCC model 32 and in an ectopic xenograft mouse model of prostate cancer 33 and lung and cervical cancers 31. If cancer cells with a hypoxic phenotype as well as cancer cells with a normoxic phenotype can be successfully targeted by specific drug regimens, as has been indicated by the data presented here, there is a possibility of improving the outcome of patients with primary liver cancer, a disease that, at present, has a dismal prognosis.

## References

[1] Parkin DM Bray F Ferlay J Pisani P Global cancer statistics, 2002. CA Cancer J Clin 2005; 55:74–108. 15761078 10.3322/canjclin.55.2.74

[2] Bruix J Sala M Llovet JM Chemoembolization for hepatocellular carcinoma. Gastroenterology 2004; 127 (Suppl 1):S179–S188. 15508083 10.1053/j.gastro.2004.09.032

[3] Ackerman NB Experimental studies on the circulation dynamics of intrahepatic tumor blood supply. Cancer 1972; 29:435–439. 5013543 10.1002/1097-0142(197202)29:2<435::aid-cncr2820290227>3.0.co;2-k

[4] Breedis C Young G The blood supply of neoplasms in the liver. Am J Pathol 1954; 30:969–977. 13197542 PMC1942491

[5] Varela M Real MI Burrel M Forner A Sala M Brunet M Chemoembolization of hepatocellular carcinoma with drug eluting beads: efficacy and doxorubicin pharmacokinetics. J Hepatol 2007; 46:474–481. 17239480 10.1016/j.jhep.2006.10.020

[6] Lammer J Malagari K Vogl T Pilleul F Denys A Watkinson A PRECISION V Investigators. Prospective randomized study of doxorubicin-eluting-bead embolization in the treatment of hepatocellular carcinoma: results of the PRECISION V study. Cardiovasc Intervent Radiol 2010; 33:41–52. 19908093 10.1007/s00270-009-9711-7 PMC2816794

[7] Lewis AL Holden RR DC Bead embolic drug-eluting bead: clinical application in the locoregional treatment of tumours. Expert Opin Drug Deliv 2011; 8:153–169. 21222553 10.1517/17425247.2011.545388

[8] Lewis AL Gonzalez MR Lloyd AW Hall B Tang Y Willis SL DC Bead: in vitro characterization of a drug-delivery device for transarterial chemoembolization. J Vasc Interv Radiol 2006; 17 (Pt 1):335–342. 16517780 10.1097/01.RVI.0000195323.46152.B3

[9] Kerbel RS Tumor angiogenesis: past, present and the near future. Carcinogenesis 2000; 21:505–515. 10688871 10.1093/carcin/21.3.505

[10] Semenza GL Hypoxia and cancer. Cancer Metastasis Rev 2007; 26:213–214. 10.1007/s10555-007-9058-y 17404692

[11] Semenza GL HIF-1, O(2), and the 3 PHDs: how animal cells signal hypoxia to the nucleus. Cell 2001; 107:1–3. 11595178 10.1016/s0092-8674(01)00518-9

[12] Lando D Peet DJ Gorman JJ Whelan DA Whitelaw ML Bruick RK FIH-1 is an asparaginyl hydroxylase enzyme that regulates the transcriptional activity of hypoxia-inducible factor. Genes Dev 2002; 16:1466–1471. 12080085 10.1101/gad.991402 PMC186346

[13] Wang B Xu H Gao ZQ Ning HF Sun YQ Cao GW Increased expression of vascular endothelial growth factor in hepatocellular carcinoma after transcatheter arterial chemoembolization. Acta Radiol 2008; 49:523–529. 18568538 10.1080/02841850801958890

[14] Li X Feng GS Zheng CS Zhuo CK Liu X Expression of plasma vascular endothelial growth factor in patients with hepatocellular carcinoma and effect of transcatheter arterial chemoembolization therapy on plasma vascular endothelial growth factor level. World J Gastroenterol 2004; 10:2878–2882. 15334691 10.3748/wjg.v10.i19.2878 PMC4572123

[15] Virmani S Rhee TK Ryu RK Sato KT Lewandowski RJ Mulcahy MF Comparison of hypoxia-inducible factor-1alpha expression before and after transcatheter arterial embolization in rabbit VX2 liver tumors. J Vasc Interv Radiol 2008; 19:1483–1489. 18922400 10.1016/j.jvir.2008.06.017 PMC2613192

[16] Semenza GL Agani F Booth G Forsythe J Iyer N Jiang BH Structural and functional analysis of hypoxia-inducible factor 1. Kidney Int 1997; 51:553–555. 9027737 10.1038/ki.1997.77

[17] Aubel-Sadron G Londos-Gagliardi D Daunorubicin and doxorubicin, anthracycline antibiotics, a physicochemical and biological review. Biochimie 1984; 66:333–352. 6380596 10.1016/0300-9084(84)90018-x

[18] Keizer HG Pinedo HM Schuurhuis GJ Joenje H Doxorubicin (adriamycin): a critical review of free radical-dependent mechanisms of cytotoxicity. Pharmacol Ther 1990; 47:219–231. 2203071 10.1016/0163-7258(90)90088-j

[19] Zunino F Capranico G DNA topoisomerase II as the primary target of anti-tumor anthracyclines. Anticancer Drug Des 1990; 5:307–317. 1963303

[20] Lee K Qian DZ Rey S Wei H Liu JO Semenza GL Anthracycline chemotherapy inhibits HIF-1 transcriptional activity and tumor-induced mobilization of circulating angiogenic cells. Proc Natl Acad Sci USA 2009; 106:2353–2358. 19168635 10.1073/pnas.0812801106 PMC2650160

[21] Sahin F Kannangai R Adegbola O Wang J Su G Torbenson M mTOR and P70 S6 kinase expression in primary liver neoplasms. Clin Cancer Res 2004; 10:8421–8425. 15623621 10.1158/1078-0432.CCR-04-0941

[22] Land SC Tee AR Hypoxia-inducible factor 1alpha is regulated by the mammalian target of rapamycin (mTOR) via an mTOR signaling motif. J Biol Chem 2007; 282:20534–20543. 17502379 10.1074/jbc.M611782200

[23] Rizell M Andersson M Cahlin C Hafström L Olausson M Lindnér P Effects of the mTOR inhibitor sirolimus in patients with hepatocellular and cholangiocellular cancer. Int J Clin Oncol 2008; 13:66–70. 18307022 10.1007/s10147-007-0733-3

[24] Wang W Jia WD Xu GL Wang ZH Li JS Ma JL Antitumoral activity of rapamycin mediated through inhibition of HIF-1alpha and VEGF in hepatocellular carcinoma. Dig Dis Sci 2009; 54:2128–2136. 19052864 10.1007/s10620-008-0605-3

[25] Wang Z Zhou J Fan J Tan CJ Qiu SJ Yu Y Sirolimus inhibits the growth and metastatic progression of hepatocellular carcinoma. J Cancer Res Clin Oncol 2009; 135:715–722. 19002496 10.1007/s00432-008-0506-z PMC12160166

[26] Wang Z Zhou J Fan J Qiu SJ Yu Y Huang XW Tang ZY Effect of rapamycin alone and in combination with sorafenib in an orthotopic model of human hepatocellular carcinoma. Clin Cancer Res 2008; 14:5124–5130. 18698030 10.1158/1078-0432.CCR-07-4774

[27] Semela D Piguet AC Kolev M Schmitter K Hlushchuk R Djonov V Vascular remodeling and antitumoral effects of mTOR inhibition in a rat model of hepatocellular carcinoma. J Hepatol 2007; 46:840–848. 17321636 10.1016/j.jhep.2006.11.021

[28] Heuer M Benkö T Cicinnati VR Kaiser GM Sotiropoulos GC Baba HA Effect of low-dose rapamycin on tumor growth in two human hepatocellular cancer cell lines. Transplant Proc 2009; 41:359–365. 19249557 10.1016/j.transproceed.2008.10.090

[29] Huynh H Chow PK Palanisamy N Salto-Tellez M Goh BC Lee CK Bevacizumab and rapamycin induce growth suppression in mouse models of hepatocellular carcinoma. J Hepatol 2008; 49:52–60. 18490075 10.1016/j.jhep.2008.02.022

[30] Zhang JF Liu JJ Lu MQ Cai CJ Yang Y Li H Rapamycin inhibits cell growth by induction of apoptosis on hepatocellular carcinoma cells in vitro. Transpl Immunol 2007; 17:162–168. 17331842 10.1016/j.trim.2006.12.003

[31] O’Reilly T McSheehy PM Wartmann M Lassota P Brandt R Lane HA Evaluation of the mTOR inhibitor, everolimus, in combination with cytotoxic antitumor agents using human tumor models in vitro and in vivo. Anticancer Drugs 2011; 22:58–78. 20890178 10.1097/CAD.0b013e3283400a20

[32] Piguet AC Semela D Keogh A Wilkens L Stroka D Stoupis C Inhibition of mTOR in combination with doxorubicin in an experimental model of hepatocellular carcinoma. J Hepatol 2008; 49:78–87. 18486258 10.1016/j.jhep.2008.03.024

[33] Grunwald V DeGraffenried L Russel D Friedrichs WE Ray RB Hidalgo M Inhibitors of mTOR reverse doxorubicin resistance conferred by PTEN status in prostate cancer cells. Cancer Res 2002; 62:6141–6145. 12414639

[34] Forster RE Tang Y Bowyer C Lloyd AW Macfarlane W Phillips GJ Lewis AL Development of a combination drug-eluting bead: towards enhanced efficacy for locoregional tumour therapies. Anticancer Drugs 2012; 23:355–369. 22241169 10.1097/CAD.0b013e32835006d2

[35] Vaupel P Kallinowski F Okunieff P Blood flow, oxygen and nutrient supply, and metabolic microenvironment of human tumors: a review. Cancer Res 1989; 49:6449–6465. 2684393

[36] Namur J Wassef M Millot JM Lewis AL Manfait M Laurent A Drug-eluting beads for liver embolization: concentration of doxorubicin in tissue and in beads in a pig model. J Vasc Interv Radiol 2010; 21:259–267. 20123210 10.1016/j.jvir.2009.10.026

[37] Namur J Citron SJ Sellers MT Dupuis MH Wassef M Manfait M Laurent A Embolization of hepatocellular carcinoma with drug-eluting beads: doxorubicin tissue concentration and distribution in patient liver explants. J Hepatol 2011; 55:1332–1338. 21703190 10.1016/j.jhep.2011.03.024

[38] Dreher MR Sharma KV Woods DL Reddy G Tang Y Pritchard WF Radiopaque drug-eluting beads for transcatheter embolotherapy: experimental study of drug penetration and coverage in swine. J Vasc Interv Radiol 2012; 23:257–264. 22178039 10.1016/j.jvir.2011.10.019 PMC3360470

[39] Sutherland RM Cell and environment interactions in tumor microregions: the multicell spheroid model. Science 1988; 240:177–184. 2451290 10.1126/science.2451290

[40] Comerford KM Wallace TJ Karhausen J Louis NA Montalto MC Colgan SP Hypoxia-inducible factor-1-dependent regulation of the multidrug resistance (MDR1) gene. Cancer Res 2002; 62:3387–3394. 12067980

[41] Ding Z Yang L Xie X Xie F Pan F Li J Expression and significance of hypoxia-inducible factor-1 alpha and MDR1/P-glycoprotein in human colon carcinoma tissue and cells. J Cancer Res Clin Oncol 2010; 136:1697–1707. 20217131 10.1007/s00432-010-0828-5 PMC2944968

[42] Zhu H Chen XP Luo SF Guan J Zhang WG Zhang BX Involvement of hypoxia-inducible factor-1-alpha in multidrug resistance induced by hypoxia in HepG2 cells. J Exp Clin Cancer Res 2005; 24:565–574. 16471319

[43] Dong Z Wang JZ Yu F Venkatachalam MA Apoptosis-resistance of hypoxic cells: multiple factors involved and a role for IAP-2. Am J Pathol 2003; 163:663–671. 12875985 10.1016/S0002-9440(10)63693-0 PMC1868200

[44] Tomida A Tsuruo T Drug resistance mediated by cellular stress response to the microenvironment of solid tumors. Anticancer Drug Des 1999; 14:169–177. 10405643

[45] Ogiso Y Tomida A Tsuruo T Nuclear localization of proteasomes participates in stress-inducible resistance of solid tumor cells to topoisomerase II-directed drugs. Cancer Res 2002; 62:5008–5012. 12208754

[46] Rharass T Vigo J Salmon JM Ribou AC New method for the detection of reactive oxygen species in anti-tumoural activity of adriamycin: a comparison between hypoxic and normoxic cells. Free Radic Res 2008; 42:124–134. 18297605 10.1080/10715760701834552

[47] Gewirtz DA A critical evaluation of the mechanisms of action proposed for the antitumor effects of the anthracycline antibiotics adriamycin and daunorubicin. Biochem Pharmacol 1999; 57:727–741. 10075079 10.1016/s0006-2952(98)00307-4

[48] McClendon AK Osheroff N DNA topoisomerase II, genotoxicity and cancer. Mutat Res 2007; 623:83–97. 17681352 10.1016/j.mrfmmm.2007.06.009 PMC2679583

[49] Potmesil M Kirschenbaum S Israel M Levin M Khetarpal VK Silber R Relationship of adramycin concentrations to the DNA lesions induced in hypoxic and euoxic L1210 cells. Cancer Res 1983; 43:3528–3533. 6861125

[50] Gerwick LE Kozin SV Stocks SJ The pH partition theory predicts the accumulation and toxicity of doxorubicin in normal and low-pH-adapted cells. Br J Cancer 1999; 79:838–842. 10070878 10.1038/sj.bjc.6690134 PMC2362684

[51] Gerwick LE Vijayappa S Kozin S Tumour pH controls the in vivo efficacy of weak acid and base chemotherapeutics. Mol Cancer Ther 2006; 5:1275–1279. 16731760 10.1158/1535-7163.MCT-06-0024

[52] Mellor HR Callaghan R Accumulation and distribution of doxorubicin in tumour spheroids: the influence of acidity and expression of P-glycoprotein. Cancer Chemother Pharmacol 2011; 68:1179–1190. 21400240 10.1007/s00280-011-1598-8

[53] Hoof T Demmer A Christians U Tümmler B Reversal of multidrug resistance in Chinese hamster ovary cells by the immunosuppressive agent rapamycin. Eur J Pharmacol 1993; 246:53–58. 7689059 10.1016/0922-4106(93)90009-x

[54] Arceci RJ Stieglitz K Bierer BE Immunosuppressants FK506 and rapamycin function as reversal agents of the multidrug resistance phenotype. Blood 1992; 80:1528–1536. 1381629

[55] Pawarode A Shukla S Minderman H Fricke SM Pinder EM O’Loughlin KL Differential effects of the immunosuppressive agents cyclosporin A, tacrolimus and sirolimus on drug transport by multidrug resistance proteins. Cancer Chemother Pharmacol 2007; 60:179–188. 17031644 10.1007/s00280-006-0357-8

